# Supplementary material for: Palmitic Acid Exerts Anti-Tumorigenic Activities by Modulating Cellular Stress and Lipid Droplet Formation in Endometrial Cancer
Source: Biomolecules. 2024 May 20;14(5):601. doi: 10.3390/biom14050601 (PMC11117634; doi:10.3390/biom14050601)
Supplement: Supplementary file 1 [file biomolecules-14-00601-s001.zip › biomolecules-2912744-supplementary.pdf]

Supplemental Figure S1

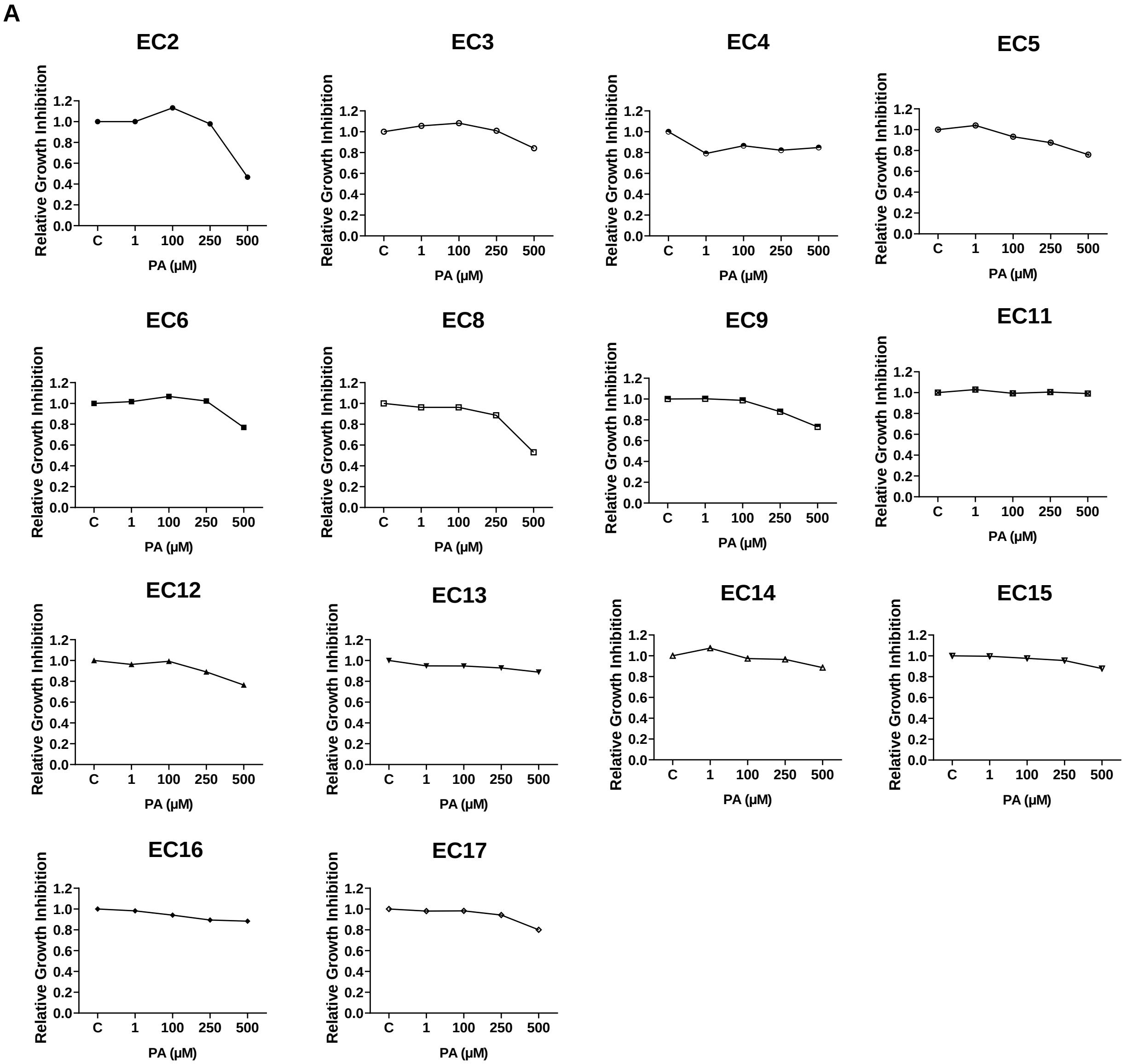

Supplemental Figure S1 Cell proliferation in the reamining14 primary cultures of human ECs treated with PA at the indicated concentrations for 72 hours (A).

Supplemental Figure S2

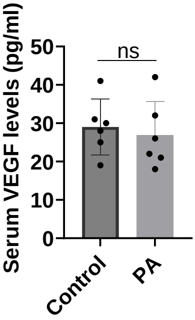

Supplemental Figure S2 Serum VEGF level in *Lkb1*$^{fl/fl}$*p53*$^{fl/fl}$ mice after PA treatment for 4 weeks

Supplemental Figure S3

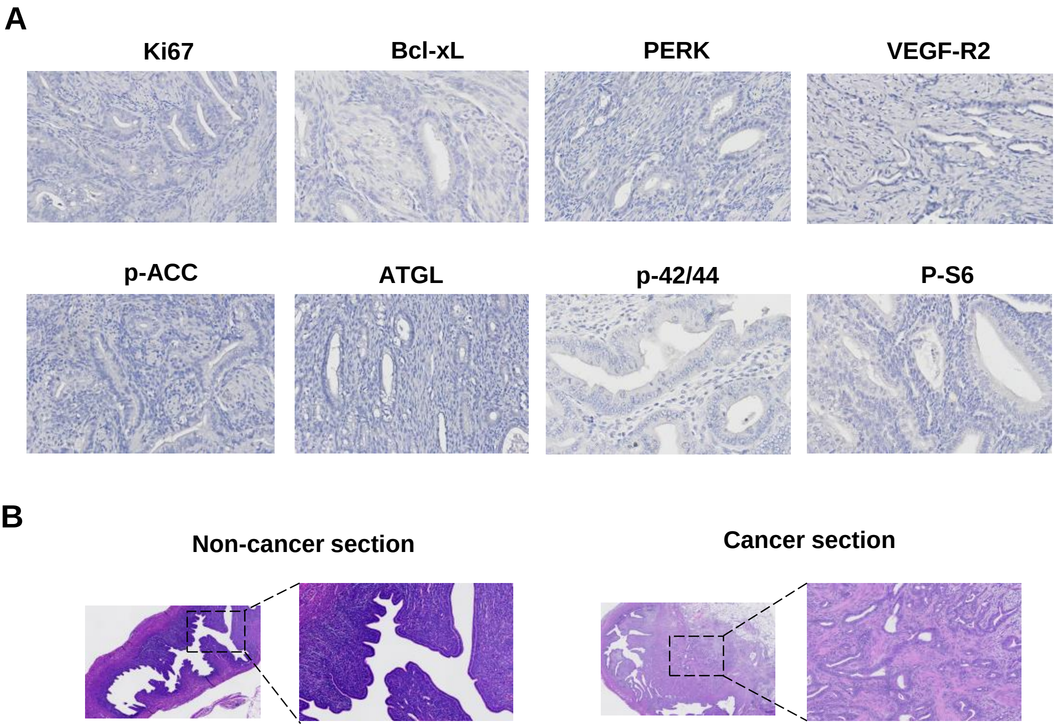

Supplemental Figure S3 Negative control slides for Ki67, Bcl-xL, PERK, VEGF-R2, p-ACC, ATGL, p-42/44, and p-S6 in EC tissues (A). H&E staining of non-cancer section and cancer section in *Lkb1*$^{fl/fl}$*p53*$^{fl/fl}$ mice (B).

Supplement table S1 Antibody used in article

Antibody	Catalogo No.	Target	Concentration
Western Blotting			
PERK	CST #5683	anti-PERK	1:1000
IRE1-α	CST #2394	anti-IRE1-α	1:1000
ATF4	CST #11815	anti-ATF4	1:1000
Bip	CST #3177	anti-Bip	1:1000
CLPP	CST #14181	anti-CLPP	1:1000
Calnexin	CST #2679	anti-Calnexin	1:1000
BCL-XL	CST #2764	anti-BCL-XL	1:1000
MCL-1	CST #5453	anti-MCL-1	1:1000
PARP	CST #9532	anti-PARP	1:1000
Snail	CST #3879	anti-Snail	1:1000
VEGF-C	CST #2445	anti-VEGF-C	1:1000
Vimentin	CST #5741	anti-Vimentin	1:1000
N-cadherin	CST #13116	anti-N-cadherin	1:1000
MMP-9	CST #13667	anti-MMP-9	1:1000
p-S6	CST #4858	anti-p-S6 (Ser235/236)	1:2000
S6	CST #2217	anti-S6	1:1000
p-Akt	CST #4060	anti-p-Akt (Ser473)	1:2000
Akt	CST #4691	anti-Akt	1:1000
p-p42/44	CST #4370	anti-p-p42/44 (Thr202/Tyr204)	1:2000
p42/44	CST #4695	anti-p42/44	1:1000
p-p38	CST #4511	anti-p-p38 (Thr180/Tyr182)	1:1000
p-4E-BP1	CST #2855	anti-p-4E-BP1 (Thr37/46)	1:1000
p-ACC	CST #11818	anti-p-ACC (Ser79)	1:1000
ATGL	CST #2439	anti-ATGL	1:1000
FASN	CST #3180	anti-FASN	1:1000
ACSL-1	CST #9189	anti-ACSL-1	1:1000
Lipin-1	CST #14906	anti-Lipin-1	1:1000
CPT1A	CST #12252	anti-CPT1A	1:1000
HK I	CST #2024	anti-HK I	1:1000
HK II	CST #2867	anti-HK II	1:1000
α -tubulin	CST #2144	anti-α -tubulin	1:1000
β-actin	CST #3700	anti-β-actin	1:1000
Glut1	Abclonal #A6982	anti-Glut1	1:1000
Glut4	Abclonal #A7637	anti-Glut4	1:1000
LDHA	Abclonal #A1146	anti-LDHA	1:1000
DGAT1	Abclonal #A6857	anti-DGAT1	1:1000
DGAT2	Abclonal #A13891	anti-DGAT2	1:1000
Immunohistochemistry			
Ki-67	CST #12202	anti-Ki-67	1:300

BCL-XL	CST #2764	anti-BCL-XL	1:1200
p-ACC	CST #11818	anti-p-ACC (Ser79)	1:500
ATGL	CST #2439	anti-ATGL	1:50
p-p42/44	CST #4370	anti-p-p42/44 (Thr202/Tyr204)	1:800
p-S6	CST #4858	anti-p-S6 (Ser235/236)	1:800
VEGF-R2	CST #9698	anti-VEGF-R2	1:1500
PERK	Santa Cruz #377400	anti-PERK	1:300
