# Supplementary figures and images for: Palmitic Acid Exerts Anti-Tumorigenic Activities by Modulating Cellular Stress and Lipid Droplet Formation in Endometrial Cancer
Source: Biomolecules. 2024 May 20;14(5):601. doi: 10.3390/biom14050601 (PMC11117634; doi:10.3390/biom14050601)

### Figure1

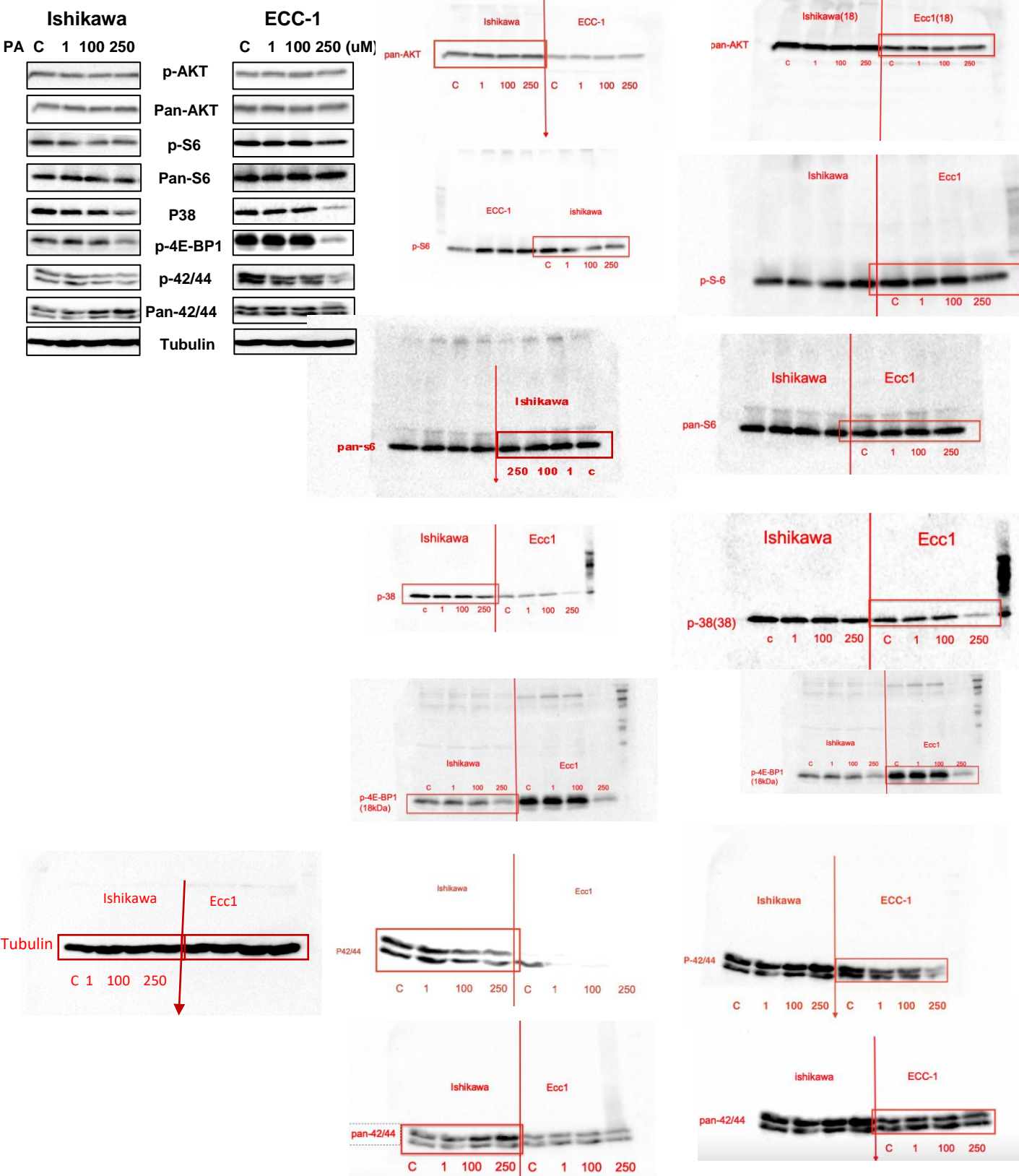

Figure2

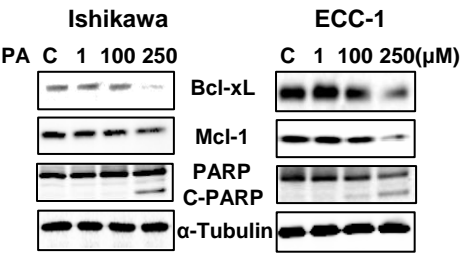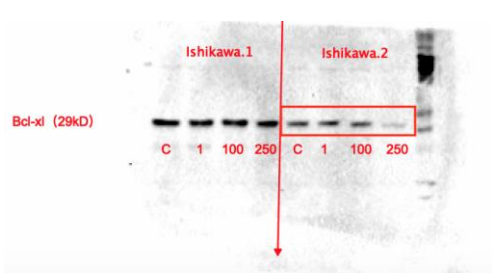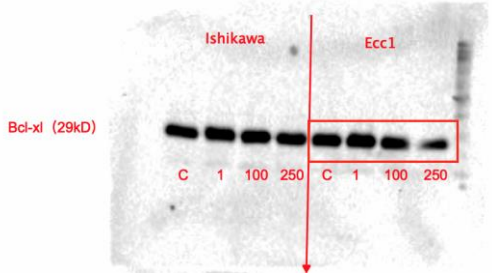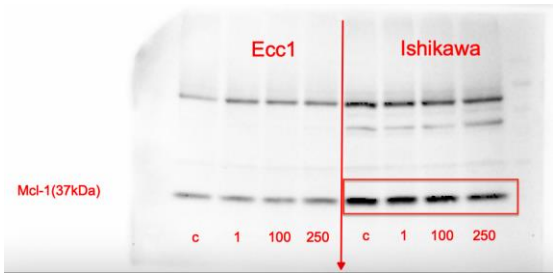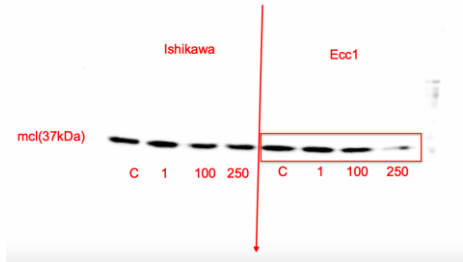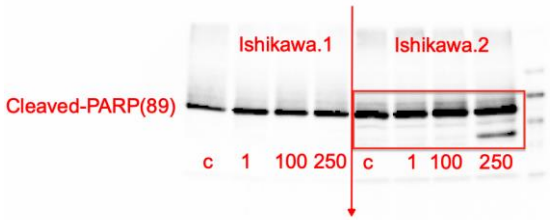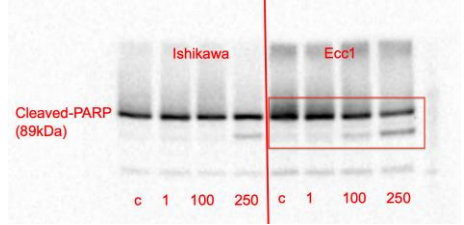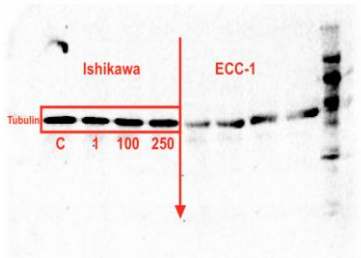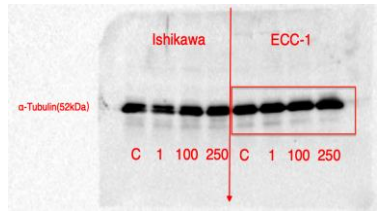

Figure3

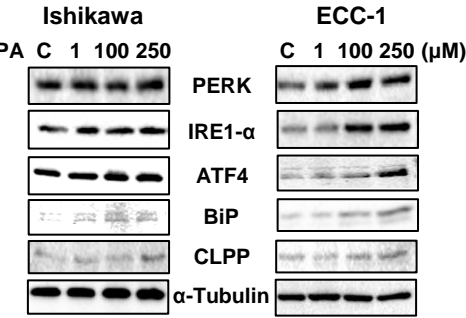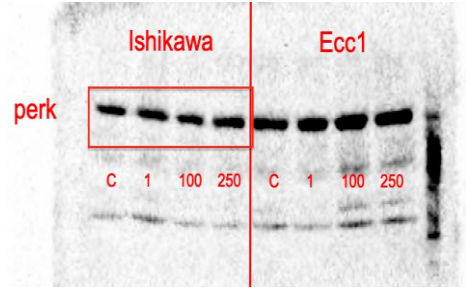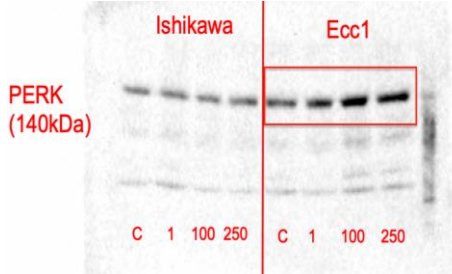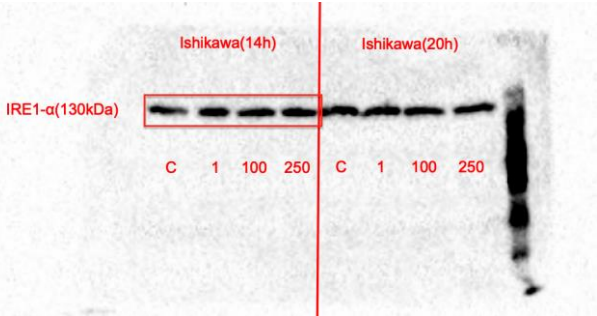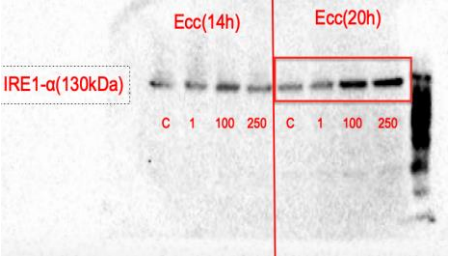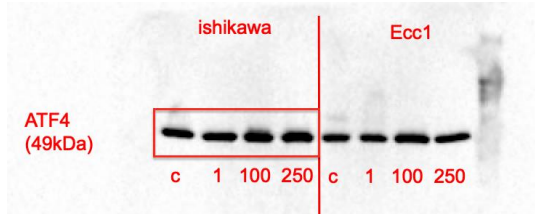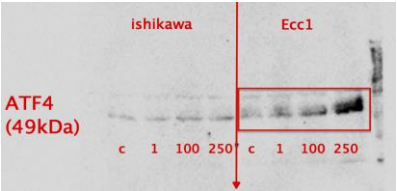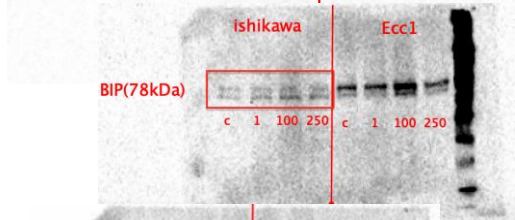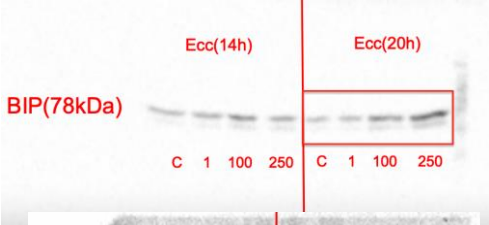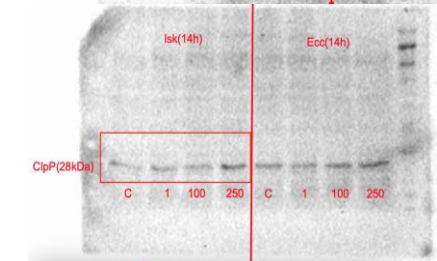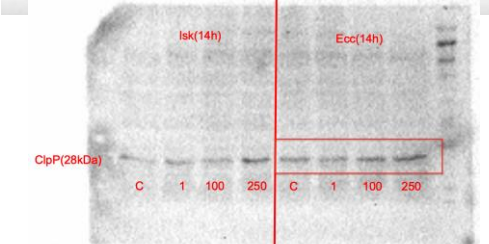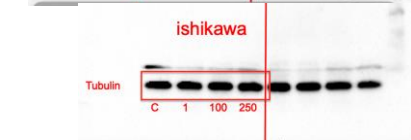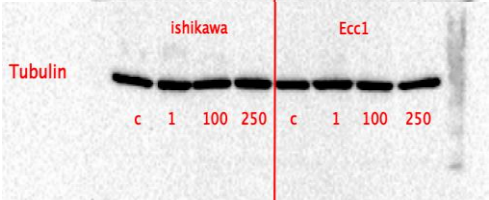

Figure4

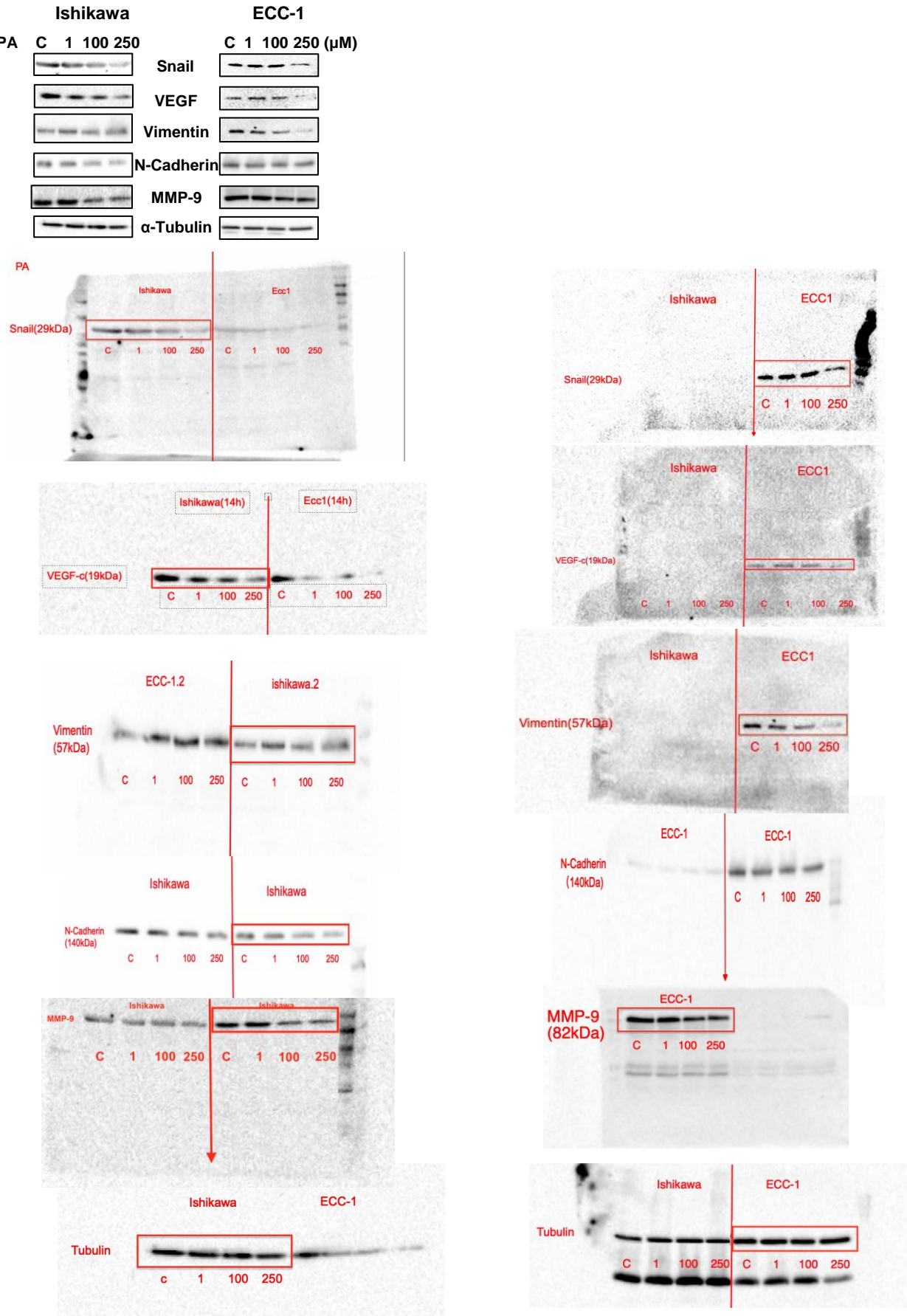

Figure5

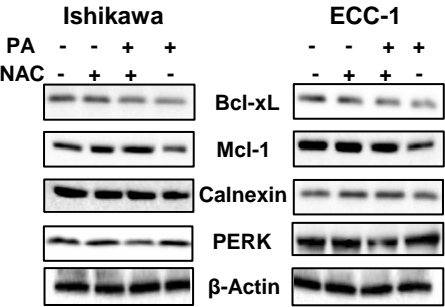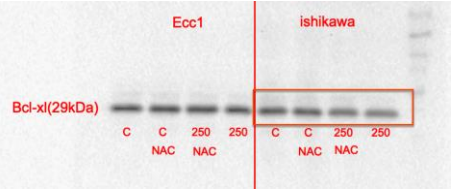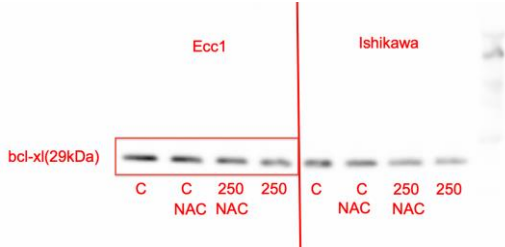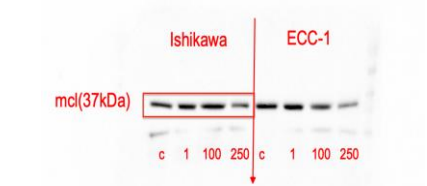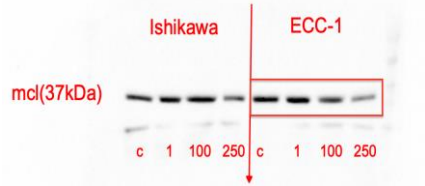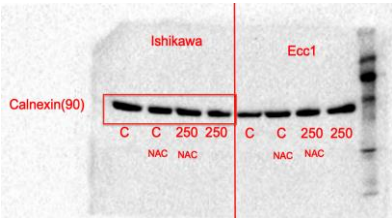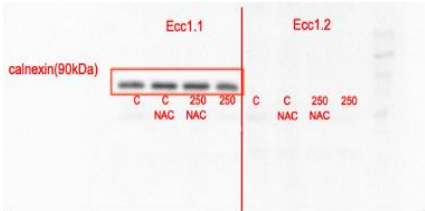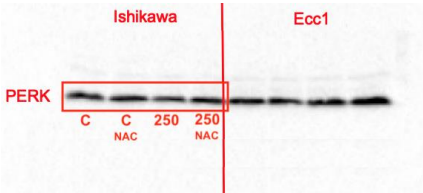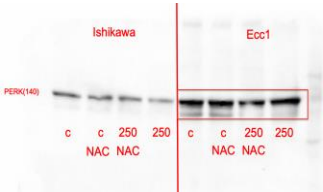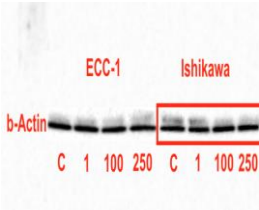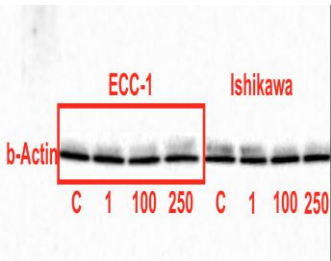

Figure6

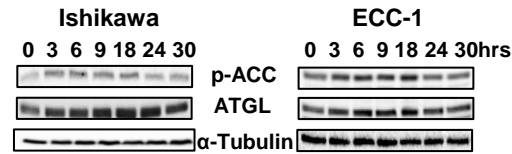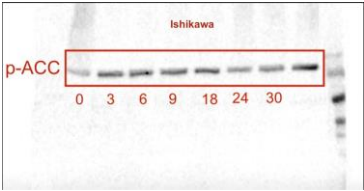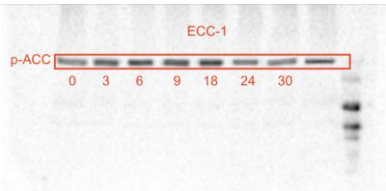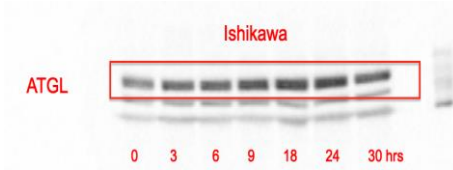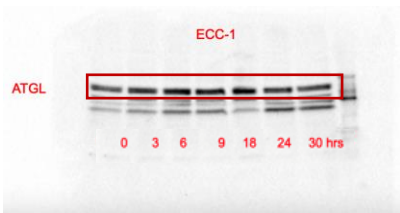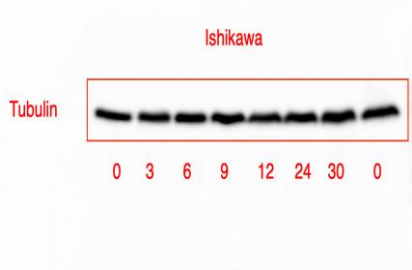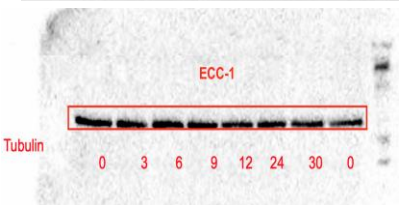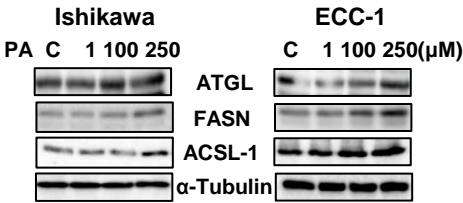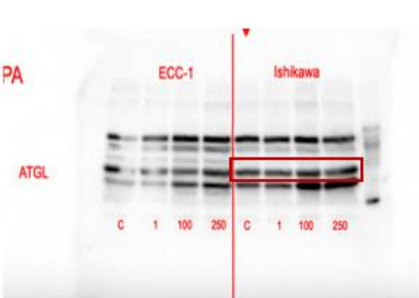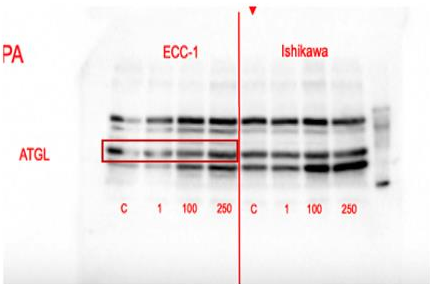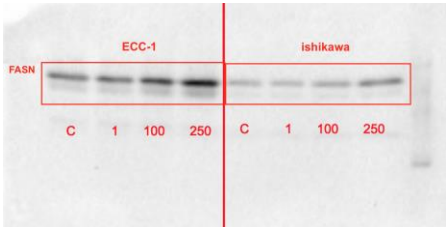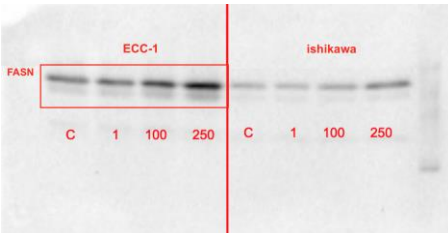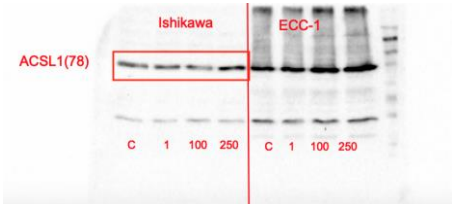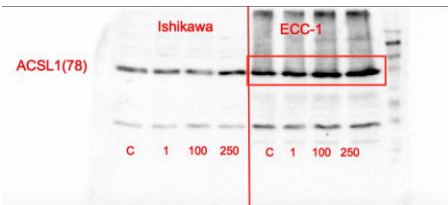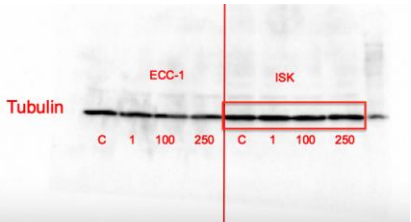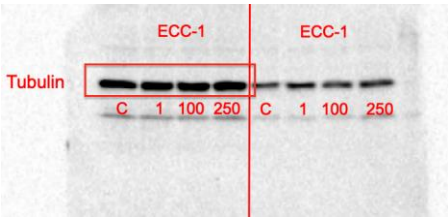

Figure6

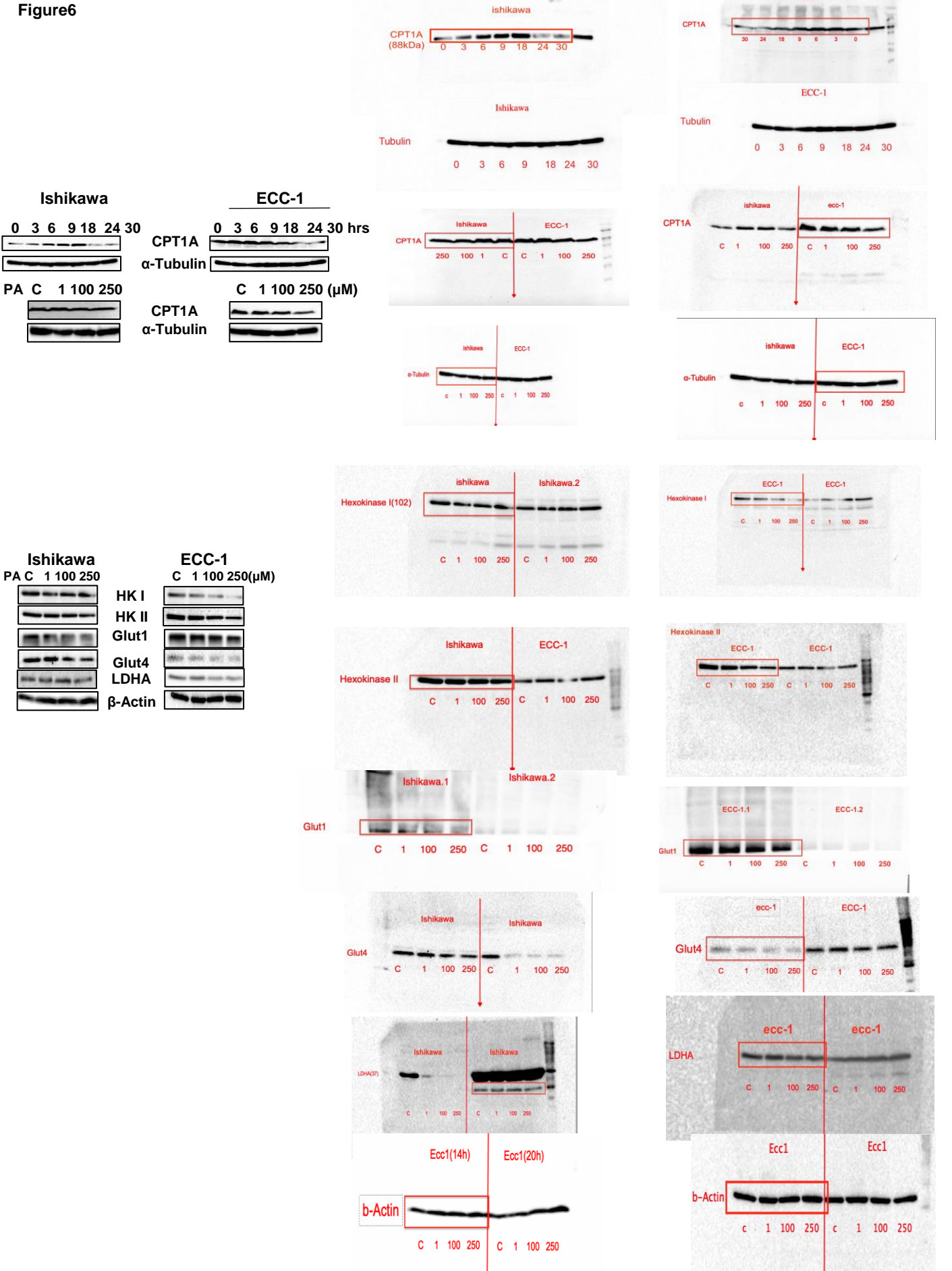

Figure7

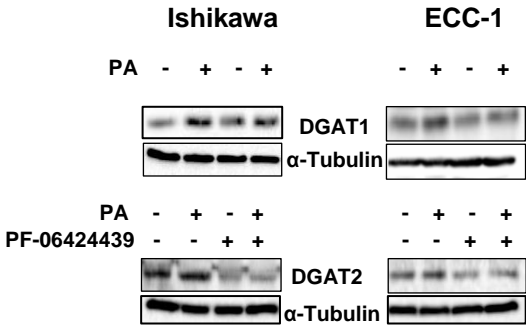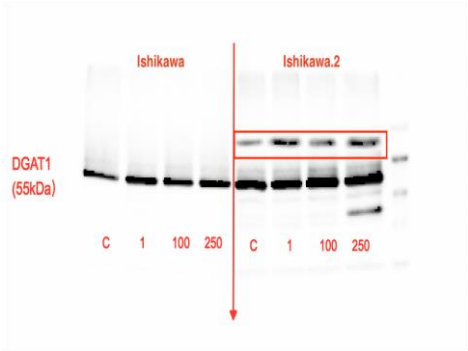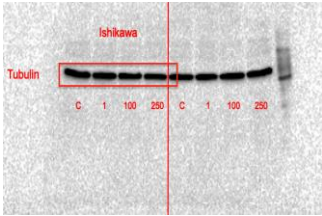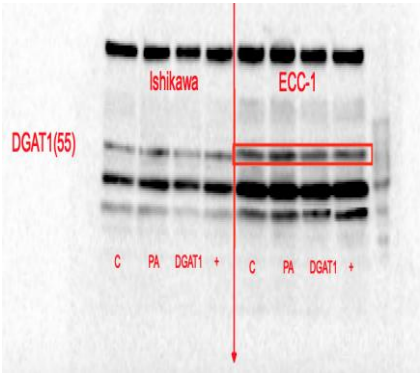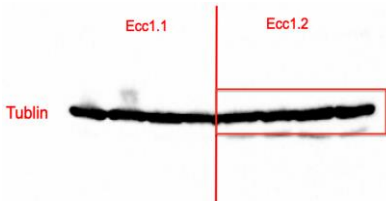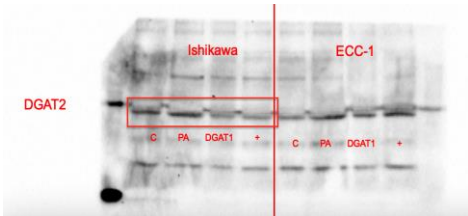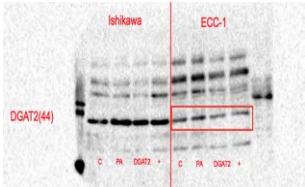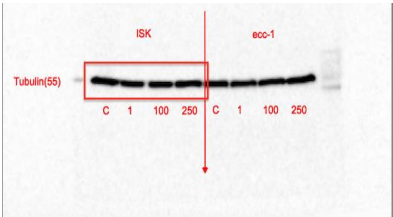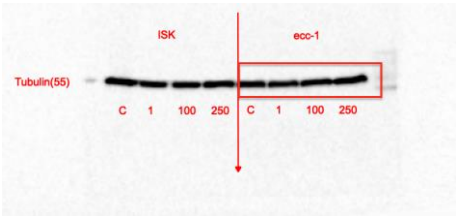

Figure8

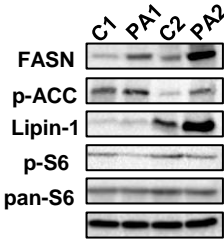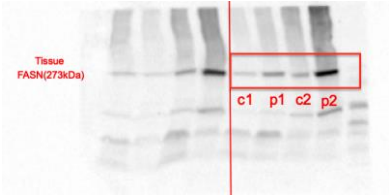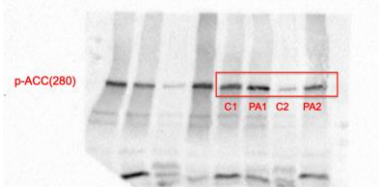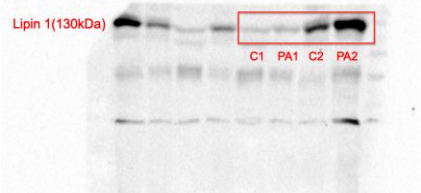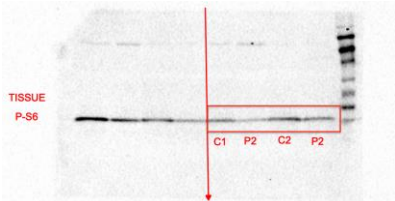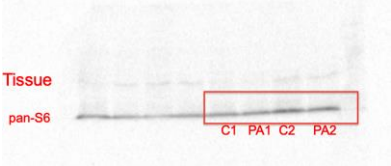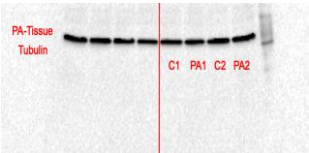

Supplement: Supplementary file 1 [file biomolecules-14-00601-s001.zip › í╛Western Blotí┐PA raw data-5.19.24.pdf]
